# The Clinical Significance of GRP78 in COVID-19 Pneumonia Across Two Regional Cohorts

**DOI:** 10.3390/ijms262110312

**Published:** 2025-10-23

**Authors:** Steliyan Petrov, Martina Bozhkova, Stanislava Popova-Belova, Mariela Geneva-Popova, Tanya Deneva, İbrahim Türkçüer, Aylin Köseler

**Affiliations:** 1 Department of Medical Microbiology and Immunology—“Prof. Dr. Elissay Yanev”, Medical University-Plovdiv, 4002 Plovdiv, Bulgaria; steliyan.petrov@mu-plovdiv.bg; 2 Department of Propedeutics of Internal Diseases “Anton Mitov”, Medical University-Plovdiv, General Hospital “Sv. Georgi”, 4002 Plovdiv, Bulgaria; stanislava.popova@mu-plovdiv.bg (S.P.-B.); mariela.geneva@mu-plovdiv.bg (M.G.-P.); 3 Department of Clinical Laboratory, Medical University-Plovdiv, 4002 Plovdiv, Bulgaria; tanya.deneva@mu-plovdiv.bg; 4 Department of Emergency, Pamukkale University Faculty of Medicine, 20190 Denizli, Türkiye; iturkcuer@pau.edu.tr; 5 Department of Biophysics, Pamukkale University Faculty of Medicine, 20190 Denizli, Türkiye; akoseler@pau.edu.tr

**Keywords:** GRP78, COVID-19, SARS-CoV-2, pneumonia, biomarkers

## Abstract

COVID-19 pneumonia remains a major driver of morbidity and mortality worldwide, with outcomes influenced by demographic, clinical, and systemic factors. Glucose-regulated protein 78 (GRP78) has emerged as a potential biomarker of disease severity, but its clinical significance across different populations remains underexplored. We conducted a retrospective cohort study including 83 hospitalized COVID-19 patients from General Hospital “Sv. Georgi”, Plovdiv, Bulgaria, and 97 from Denizli, Türkiye. Pneumonia diagnosis was based on clinical, laboratory, and radiological criteria. Patient demographics and biomarkers (CRP, D-dimer, oxygen saturation, lymphocyte count, and serum GRP78) were compared between cohorts. Statistical analyses included Mann–Whitney U, *t*-tests, and logistic regression. Pneumonia prevalence was 48.8% in patients from General Hospital “Sv. Georgi”, Plovdiv and 58.8% in Denizli. Patients in Plovdiv were older and exhibited higher CRP, D-dimer, and GRP78 levels, alongside lower oxygen saturation and lymphocyte counts. Logistic regression demonstrated that GRP78 significantly predicted pneumonia in both cohorts, with robust discriminative performance on ROC analysis. This study highlights significant regional differences in COVID-19 pneumonia presentation between Bulgaria and Türkiye. Elevated GRP78 levels were strongly associated with pneumonia and other severity markers, underscoring its potential as a clinically valuable biomarker for early risk stratification. These findings emphasize the importance of both localized epidemiological analyses and biomarker-based approaches to optimize COVID-19 management.

## 1. Introduction

Coronavirus disease 2019 (COVID-19), caused by the novel Severe Acute Respiratory Syndrome Coronavirus 2 (SARS-CoV-2), continues to impose a substantial global health burden [1]. While the clinical spectrum of COVID-19 ranges from asymptomatic infection to critical illness, pneumonia has emerged as one of the most frequent and severe complications, often leading to respiratory failure, Intensive Care Unit (ICU) admission, and increased mortality [2]. The burden of COVID-19 pneumonia is not distributed evenly across populations; rather, it is shaped by a complex interplay of individual risk factors and systemic variables, including age, sex, comorbidities, healthcare infrastructure, and regional public health responses [3].

Previous research has demonstrated that elderly individuals and patients with underlying conditions such as hypertension, diabetes, or chronic respiratory diseases are at heightened risk of developing severe pneumonia following SARS-CoV-2 infection [4,5]. In addition, diagnostic confirmation of COVID-19 pneumonia typically relies on chest imaging modalities, such as chest X-ray and computed tomography, which can reveal characteristic findings like bilateral ground-glass opacities, consolidation, or interstitial changes [1]. Laboratory tests and pulse oximetry further aid in assessing disease progression and severity [1]. Risk factors for the occurrence of pneumonia in COVID-19 patients extend beyond advanced age and comorbidities, encompassing immunosuppression, obesity, delayed hospital admission, and elevated viral load at presentation [1,2]. Furthermore, variations in diagnostic capabilities and treatment practices may influence clinical outcomes across regions. Biomarkers such as C-reactive protein (CRP), D-dimer, lymphocyte count, and emerging indicators like glucose-regulated protein 78 (GRP78) have been linked to disease severity and may provide insight into systemic inflammatory responses associated with COVID-19 pneumonia [6,7,8,9,10].

GRP78, an endoplasmic reticulum stress protein, functions as a molecular chaperone that is upregulated under conditions of cellular stress and viral infection [7]. In the context of COVID-19, elevated serum GRP78 levels have been associated with severe disease, possibly reflecting heightened inflammatory and metabolic stress responses [8]. Moreover, GRP78 has been suggested to facilitate viral entry and propagation, highlighting its potential dual role as both a biomarker and a therapeutic target [6].

The heterogeneity in disease presentation and severity across different regions underscores the importance of localized epidemiological studies. In particular, comparative analyses between countries with distinct healthcare systems and sociocultural contexts can offer valuable information about the factors driving disease burden and outcomes. This study seeks to address this need by comparing the prevalence of pneumonia among hospitalized COVID-19 patients in two distinct geographic regions: Plovdiv, Bulgaria; and Denizli, Türkiye.

In addition to identifying the frequency of pneumonia in these populations, this study aims to examine differences in patient demographics and key clinical biomarkers, including CRP, D-dimer, oxygen saturation, serum GRP78, and lymphocyte levels. By identifying patterns and discrepancies in clinical presentation and laboratory indicators, this research seeks to contribute to a more nuanced understanding of how regional and systemic factors may influence COVID-19 pneumonia outcomes. Such knowledge can support the development of targeted strategies for early intervention, optimized treatment, and resource allocation tailored to local healthcare settings.

## 2. Results

In Plovdiv, 40 patients were diagnosed with pneumonia. In Denizli, 57 out of 97 patients were diagnosed with pneumonia. The prevalence of pneumonia was higher in Denizli compared to Plovdiv. The male to female ratio of pneumonia-positive patients was comparable between the two cohorts (Plovdiv: 1.5; Denizli: 1.48). Information concerning sex, presence of pneumonia, and disease severity is presented in Table 1.

**Figure 1:**
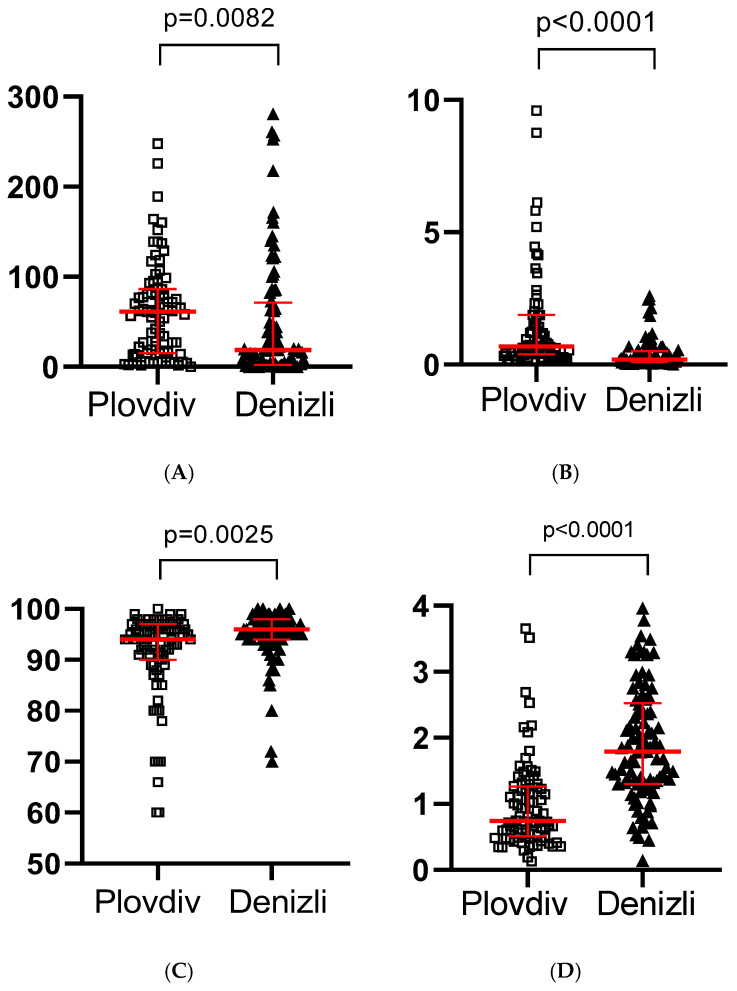
(**A**)—Dot plot from a Mann–Whitney U test comparing serum CRP levels (mg/L) between the two patient groups from Plovdiv and Denizli (Median 61.5 mg/L (0.00–395.00) vs. 18.79 mg/L (0.00–359.50), 95%CI −35.97, −4.73). (**B**)—Dot plot from a Mann–Whitney U test comparing serum D-dimer levels (mg/L) between the two patient groups from Plovdiv and Denizli (Median: 0.68 mg/L (0.17–35.20) vs. 0.18 mg/L (0.01–2.59), 95% CI −0.60, −0.29). (**C**)—Dot plot from a Mann–Whitney U test comparing oxygen saturation levels (%) between the two patient groups from Plovdiv and Denizli (Median: 94.00% (60.00–100) vs. 96.00% (70.00–100), 95% CI 1, 3). (**D**)—Dot plot from a Mann–Whitney U test comparing lymphocyte counts (×10^9^/L) between the two patient groups from Plovdiv and Denizli (Median: 0.74 × 10^9^/L (0.13–3.65) vs. 1.79 × 10^9^/L (0.14–5.50), 95% CI 0.67, 1.09).

Plovdiv patients exhibited higher CRP levels compared to those observed in Denizli, as shown in Figure 1A.

D-dimer levels were markedly elevated in Plovdiv, as shown in Figure 1B. Oxygen saturation was found to be lower in Plovdiv compared to Denizli, as depicted in Figure 1C. Significantly lower lymphocyte counts were discovered in Plovdiv compared to Denizli, as presented in Figure 1D.

**Figure 2:**
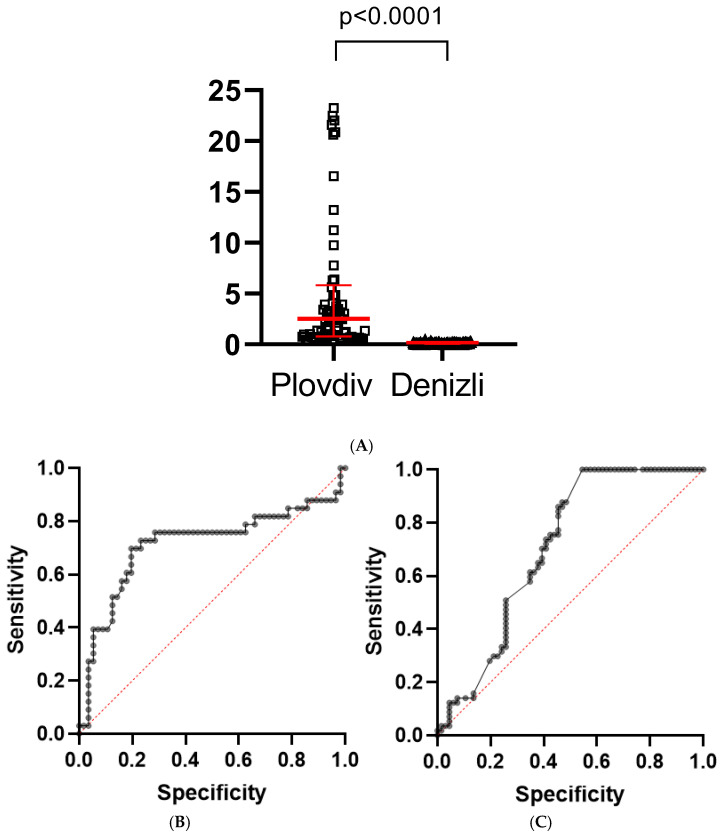
(**A**)—Dot plot from a Mann–Whitney U test comparing serum GRP78 levels (pg/mL) between the two patient groups from Plovdiv and Denizli (Median: 2.50 pg/mL (0.39–64.42) vs. 0.13 pg/mL (0.06–0.5), 95% CI −2.88, −1.28). (**B**)—Receiver operating characteristic (ROC) curve of GRP78 serum levels for predicting pneumonia in Plovdiv patients’ group. The ROC curve shows that GRP78 serum levels discriminate pneumonia cases from controls with good accuracy, lying above the reference line (red). The area under the curve (AUC) indicates good predictive performance (0.714, 95% CI 0.588, 0.840); (**C**)—ROC curve of GRP78 serum levels for predicting pneumonia in Denizli patients’ group. The ROC curve shows that GRP78 serum levels discriminate against pneumonia cases from controls with good accuracy (0.705, 95% CI 0.612, 0.798), lying above the reference line. The AUC indicates good predictive performance.

When assessing the GRP78 levels, it was found that their values were dramatically higher in Plovdiv serum samples, compared to Denizli’s results. Results are presented in Figure 2A.

Results from the logistic regression analysis show that GRP78 serum levels can predict the development of pneumonia in both groups (Figure 2B,C).

**Figure 3:**
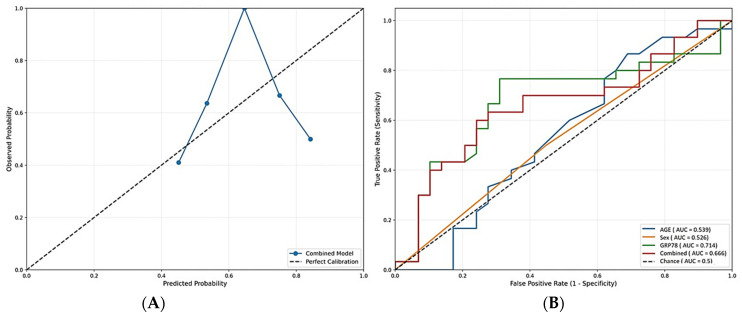
(**A**)—Calibration plot demonstrating good agreement between predicted and observed risks across deciles of predicted probability; (**B**)—ROC analysis demonstrating good discrimination for GRP78 levels alone, poor discrimination for the other predictors alone, and all three combined.

Age, sex, and comorbidities (low % occurrence) were not significantly associated with pneumonia/severity in univariate analysis. Evaluation of Model Calibration and Discrimination is presented in Table 2 and Figure 3A,B. The Hosmer–Lemeshow test showed results with a *p*-value of 0.677, meaning that the test indicates good model calibration (Figure 3A). The model demonstrated poor discrimination of pneumonia using age and sex as single predictors (Figure 3B). Serum GRP78 levels showed the highest AUC value as a single predictor. When adding the other predictors to the model, the AUC dropped, showcasing that the combined model brings more noise to the overall result. At the optimal threshold of 0.491 (determined by Youden’s index), Sensitivity was 60.0% (correctly identified 60% of pneumonia cases) and Specificity was at 75.9% (correctly identified 76% of non-pneumonia cases). 

## 3. Discussion

The broader role of GRP78 extends to multiple other pathological conditions. In oncology, GRP78 is consistently overexpressed in tumors, where it contributes to cancer cell survival, resistance to chemotherapy, and poor prognosis, thus being pursued as a therapeutic target with antibodies, peptides, and small molecules [11]. In metabolic disorders such as diabetes and obesity, sustained endoplasmic reticulum stress and GRP78 upregulation exacerbate inflammation and insulin resistance [12]. Additionally, GRP78 functions as a co-receptor for other viruses, including Japanese encephalitis and Zika viruses, underscoring its importance as a general viral entry mediator [13]. This duality means that it is pro-pathogenic in viral and oncological contexts, yet potentially protective in neurodegeneration, illustrating the complexity of GRP78 as both a biomarker and a therapeutic target, depending on the disease environment.

In relation to SARS-CoV-2, GRP78 has been identified as a potentially important contributor to the infection process. Cell-surface-localized GRP78 (csGRP78), which appears under endoplasmic reticulum stress, can directly interact with the viral spike protein and facilitate viral entry in cooperation with ACE2. Preclinical studies have demonstrated that targeting csGRP78 with monoclonal antibodies (such as hMAb159) or small-molecule inhibitors (including YUM70 and HA15) reduces spike-mediated viral entry, replication, and associated lung injury [14,15]. Moreover, synergistic effects have been observed when GRP78 inhibitors are combined with established antivirals such as Nirmatrelvir [16]. These findings highlight the dual significance of GRP78 in COVID-19, not only as a circulating biomarker of severity, but also as a potential therapeutic target that could be exploited in adjunctive antiviral strategies.

The extent of pneumonia among patients with COVID-19 from Plovdiv, Bulgaria, and Denizli, Türkiye, is strongly indicative of regional differences that are connected throughout the patient demographic. The role of age, sex, and comorbidities is very important in understanding the variation in the distribution of the disease, clearly shown in the reports of Altunok et al. [17] and Kostadinova et al. [18]. They illustrate that the elderly, particularly those with underlying health conditions, make up a group at high risk for the development of severe pneumonia during COVID-19 infections.

Older adults who are in their prime with comorbidities are typically the group that is most greatly affected. Here, the most prevalent comorbidities are hypertension, diabetes, and chronic respiratory diseases. Such people are particularly vulnerable to COVID-19, and thus are most prone to having respiratory complications caused by the virus [4,5]. Furthermore, demographic factors are found to be the ones which most significantly foresee the severity of pneumonia and later health results in COVID-19 patients. In fact, age stratification makes it clear that individuals 65 years or older represent a greater portion of hospitalized cases and, moreover, have higher rates of pneumonia-related morbidity and mortality in both regions [19].

Gender differences are outlined as relevant, with men customarily presenting more serious effects of pneumonia caused by COVID-19 that may be due to biological [3,20,21], behavioral, and socioeconomic factors [22,23], which are cited as significant causes of pneumonia and/or death incidence in the context of COVID-19. The co-existence of these gender configurations with the age factor and comorbidity is the key aspect of a proper interpretation of the epidemiological landscape of pneumonia in these two different cultural and health environments.

Significantly, these characteristics in the population certainly make it clear that health campaigns have to be changed to suit the needs of high-risk groups in the most effective way. In Bulgaria and Türkiye, where health systems have high variance in terms of availability and access to resources, recognition of the implications of these demographic factors on the prevalence of pneumonia can empower policy formulators with essential information.

In addition, there are also differences between the health systems’ capabilities and responses in Plovdiv and Denizli that should be acknowledged, as they can bring about increased vulnerabilities in populations at risk. The efficiency of each region that can respond to the pneumonia load associated with COVID-19 is affected by variations in health accessibility, availability of intensive care resources, and public health initiatives. The wise distribution of resources is thus based on new knowledge of these systemic variations.

Such interdependencies demonstrate the importance of collecting and analyzing comprehensive patient data, resources for healthcare, and clinical results. This information may lead to the introduction of targeted preventive measures, as well as the enhancement of capacity in the management of the pneumonia caused by COVID-19. The direct correlation between demographic risks and clinical results, therefore, provides us with a very important research area in which new studies must be carried out. These should be aimed at improving interventions in the provision of services to vulnerable populations in Plovdiv and Denizli. Clinical indicators like inflammation markers, respiratory difficulties, and the necessity for mechanical ventilation are a few examples of the essential areas that will help us understand the severity of pneumonia in COVID-19 patients.

A cross-sectional comparison between Plovdiv, Bulgaria, and Denizli, Türkiye, reveals that the levels of these indicators differ substantially. The reasons for such differences might be in-patient demographics, clinical practices, and the availability of health system resources. PDW and CRP (platelet distribution width and C-reactive protein) were shown to be very good prognosis markers for severe pneumonia in COVID-19 in reports from both regions [24,25]. To be specific, high PDW levels were related to exacerbated inflammation in the literature; therefore, it is reasonable to expect that patients in these regions, where the highest prevalence of PDW is observed, could be the ones to experience the most severe courses of the disease.

The observed higher GRP78 levels in the Plovdiv cohort, which also presented with a higher burden of severe clinical indicators, strongly suggests that GRP78 may serve as a valuable biomarker for identifying patients at risk of more severe COVID-19 outcomes, including pneumonia. GRP78 has been increasingly recognized for its role in unfolded protein response and cellular stress, which are critically involved in viral infections, including SARS-CoV-2 [26,27]. The significant elevation of GRP78 in patients with more pronounced inflammation (higher CRP), coagulopathy (higher D-dimer), and respiratory compromise (lower oxygen saturation) reinforces its potential as a clinically relevant biomarker.

While factors like age, sex, and comorbidities are known to influence COVID-19 outcomes, and variations in healthcare systems and public health responses can impact disease presentation and severity, the consistent association of elevated GRP78 with a more severe clinical profile across both cohorts is particularly noteworthy [7,28]. The differences observed between Plovdiv and Denizli, rather than diminishing the significance of GRP78, may in fact strengthen its role as a consistent indicator of disease severity, even within diverse patient populations. The fact that GRP78 levels align with other established markers of severity, despite the confounding variables inherent in a multi-center study, further supports its potential as a reliable biomarker. While cross-cohort differences in GRP78 were observed, these should be considered exploratory due to methodological differences between sites. The primary evidence supporting GRP78 as a predictor comes from within-cohort analyses and ROC performance.

Of similar importance is the awareness of the ways in which health disparities could become the root causes of systemic inequalities in health results. Stakeholders in alleging health policy formulators, doctors, and public health advocates, amongst others, can help create personalized health strategies that improve pneumonia management in COVID-19 patients in both regions by recognizing and comprehending these disparities. It is important to address gaps in healthcare, ensure that medical resources are accessible, and build public trust in vaccination campaigns, which are essential steps to reduce the burden of the disease caused by COVID-19 pneumonia.

Future research should focus on further elucidating the precise mechanisms by which GRP78 levels correlate with COVID-19 severity and exploring its potential for prognostic value and guiding treatment strategies. Longitudinal studies tracking GRP78 levels over the course of the disease and their correlation with patient outcomes such as ICU admission, need for mechanical ventilation, and mortality would provide valuable insights. Additionally, investigating GRP78 in a larger and more diverse patient population would help to validate its utility across different demographic and clinical settings.

## 4. Materials and Methods

Study Design and Population: A retrospective cohort study was conducted, including data from 83 COVID-19 patients hospitalized at “St. George” General hospital in Plovdiv, Bulgaria, and 97 COVID-19 patients hospitalized at Pamukkale university hospital in Denizli, Türkiye. Patients were selected based on confirmed SARS-CoV-2 infection using RT-PCR testing. This study was approved by the institution’s Ethics committee.

Data Collection: Clinical records were reviewed to identify the presence of pneumonia. Pneumonia was diagnosed based on clinical symptoms, laboratory findings, and radiological imaging. COVID-19 severity was evaluated according to the criteria established by the World Health Organization. Additional data collected included age, CRP, D-dimer, oxygen saturation, and serum GRP78 and lymphocyte levels.

Methods: In the Bulgarian cohort, serum levels of GRP78 were assessed via an enzyme-linked immunosorbent assay (Gentaur, Kampenhout, Belgium, # E01G0368); D-dimer concentration was determined in citrate plasma based on the immunoturbidimetric principle on the Sysmex coagulometer CS 2000, Siemens Healthcare (Tokyo, Japan). Serum levels of CRP were determined using a turbidimetric assay. The tests were carried out using Beckman Coulter reagents on an automated clinical chemistry analyzer Olympus AU 480, Beckman Coulter, Inc., Brea, CA, USA, according to original programs.

In the Turkish cohort, serum levels of GRP78 were assessed via an enzyme-linked immunosorbent assay (Human Glucose Regulated Protein 78 (GRP78) ELISA Kit, Sun Long, SL2048Hu, Hangzhou City, China). Additional data collected included age, CRP, D-dimer, and lymphocytes.

Due to differences in the assay kits and cohort characteristics, comparisons of absolute GRP78 levels between Plovdiv and Denizli should be interpreted as exploratory.

Statistical Analysis: All data were entered into the GraphPad Prism ver.8.0. software and subjected to statistical evaluation. Clinical characteristics and laboratory parameters were compared between patients using the Mann–Whitney U or the *t*-test where suitable. The existence of a normal distribution was investigated using the One-Sample Kolmogorov–Smirnov test, and is presented as median (min–max). The prevalence of pneumonia was calculated as the proportion of patients diagnosed with pneumonia in each cohort. Statistical significance was set at *p* < 0.05. We built a multivariable logistic regression model using the available biomarkers to predict the probability of pneumonia, and evaluated discrimination using ROC/AUC.

## 5. Conclusions

In conclusion, this study demonstrates significant regional differences in the prevalence and clinical presentation of COVID-19 pneumonia between Plovdiv, Bulgaria, and Denizli, Türkiye, underscoring the importance of localized analyses to guide targeted public health interventions and optimize patient care. At the same time, our findings provide compelling evidence for the potential of GRP78 as a valuable biomarker of disease severity, with its elevation showing a striking association with other indicators of severe COVID-19, particularly in the Plovdiv cohort, which experienced a higher overall disease burden. Together, these results highlight both the need for regional strategies in managing COVID-19 and the promise of GRP78 for identifying high-risk patients. Future research should further explore the underlying drivers of these disparities and fully establish the prognostic and diagnostic value of GRP78 in COVID-19 management.

## 6. Limitations

This study has some limitations that should be acknowledged. First, there were demographic differences between the Plovdiv and Denizli cohorts, particularly in terms of age distribution and sex ratio. However, these differences are unlikely to have introduced major bias into our findings, as no significant statistical associations were observed between sex and the presence of pneumonia, sex and disease severity, or age and pneumonia/severity outcomes. Therefore, while demographic variability exists between the groups, it does not appear to confound the main results of this study. Although different ELISA kits are used for the evaluation of serum GRP78 levels, the ROC results confirm the predictive capability in both cohorts. Nevertheless, results should be considered exploratory.

## Figures and Tables

**Table 1:** Data from COVID-19 patients from Plovdiv, Bulgaria, and Denizli, Türkiye, showing distribution based on sex, presence of pneumonia, disease severity, and comorbidities.

Column1	Plovdiv	Denizli	*p* Value
Patients (N)	82	97	
Males (N, %)	42 (51.22%)	58 (59.79%)	0.2497
Females (N, %)	40 (48.78%)	39 (40.20%)	0.2497
Age (Median, range)	61 (32–86)	48 (18–88)	0.0012
Pneumonia (N, %)	40 (48.78%)	57 (58.76%)	0.1816
Moderate COVID-19 (N, %)	45 (54.88%)	73 (75.26%)	0.0042
Severe COVID-19 (N, %)	37 (45.12%)	24 (24.74%)	0.0042
Hypertension (N, %)	13 (15.85)	16 (16.49)	
Diabetes mellitus (N, %)	6 (7.31)	15 (15.46)	

**Table 2:** Multivariable logistic regression table detailing the odds ratios, 95% confidence intervals, *p*-values, a list of all variables included, and the events per variable ratio.

Variable	Events per Variable Ratio	Coefficient (β)	Odds Ratio (OR)	95% CI	*p*-Value of Likelihood Ratio Test
Age	13.3	−0.0048	0.9952	0.96–1.032	0.797
sex	13.3	−0.2345	0.791	0.277–2.259	0.6615
Serum GRP78 levels	13.3	0.0637	1.066	1.017–1.137	0.0055

## Data Availability

The raw data supporting the conclusions of this article will be made available by the authors on request.

## Abbreviations

The following abbreviations are used in this manuscript:

COVID-19	Coronavirus disease 2019
SARS-CoV-2	Severe Acute Respiratory Syndrome Coronavirus 2
GRP78	Glucose-regulated protein 78
CRP	C-reactive protein
ICU	Intensive Care Unit
